# Prenatal Diagnosis of Fragile X: Can a Full Mutation Allele in the *FMR1* Gene Contract to a Normal Size?

**DOI:** 10.3389/fgene.2017.00158

**Published:** 2017-11-03

**Authors:** Esther Manor, Azhar Jabareen, Nurit Magal, Arei Kofman, Randi J. Hagerman, Flora Tassone

**Affiliations:** ^1^Faculty of Health Sciences, Ben-Gurion University of the Negev, Beersheba, Israel; ^2^Genetics Institute, Soroka Medical Center, Beersheba, Israel; ^3^Department of Medical Genetics, Rabin Medical Center, Petah Tikva, Israel; ^4^Felsenstein Medical Research Center, Rabin Medical Center, Petah Tikva, Israel; ^5^Department of Pediatrics, MIND Institute, UC Davis Medical Center, Sacramento, CA, United States; ^6^Department of Biochemistry and Molecular Medicine, School of Medicine, University of California, Davis, Davis, CA, United States; ^7^MIND Institute, UC Davis Medical Center, Sacramento, CA, United States

**Keywords:** prenatal diagnosis, *FMR1* gene, instability, expansion, contraction

## Abstract

Here we describe a case of a prenatal diagnosis of a male fetus that inherited the unstable allele from his full mutation mosaic mother (29, 160, >200 CGG repeats) reduced to a normal size range (19 CGG repeats). Haplotype analysis showed that the fetus 19 CGG repeats allele derived from the maternal unstable allele which was inherited from his maternal grandmother. No size mosaicism was detected by testing the DNA from *in vitro* cultured samples, including seventh passage culture as well as from two amniocentesis samples. Sequence analysis confirmed that the allele was 19 CGG repeats long. Methylation assay showed no methylation. Although none of the techniques used in this study can provide with absolute certainty the diagnosis, the results strongly indicate the presence in the fetus of an allele with a CGG repeat number in the normal range. Because this is a prenatal diagnosis case, the crucial question is whether the 19 CGG allele derived from the maternal unstable expanded allele, which contracted to the normal range, became a normal stable allele or a normal unstable allele which could expand in the next generation. It is also possible that allele size mosaicism of the *FMR1* gene that went undetected exists. Because this is a prenatal diagnosis case, we cannot with certainty exclude the presence of an undetected expanded allele of the *FMR1* gene, in addition to the 19 CGG allele derived from an unstable expanded allele, which contracted to the normal range.

## Introduction

Fragile X syndrome (FXS) is the most common inherited cause of intellectual disability and the most common monogenic cause of Autism Spectrum Disorders (Harris et al., 2008). The FXS is caused by a dynamic mutation in the *FMR1* gene, which is located at Xq27.3. A normal gene carries up to 54 CGG repeats in the 5′ UTR while a premutation allele carries 55–200 CGG repeats and is considered unstable during transmission to offspring. The full mutation allele harbors more than 200 CGG repeats and shows somatic mosaicism in 12–41% cases (Nolin et al., 1994; Rousseau et al., 1994; Merenstein et al., 1996). Full mutation alleles are usually methylated and transcriptionally silent so that no *FMR1* protein (FMRP) is produced (Kremer et al., 1991; Oberle et al., 1991; Yu et al., 1991; Verkerk et al., 1993; Allingham-Hawkins et al., 1999). Almost 100% of the males who inherited a full mutation show FXS that usually involves intellectual disability, while only about 60% of the females with a full mutation show mild to severe clinical features. It is generally accepted that the *FMR1* instability occurs when the CGG repeats expanded beyond 55 CGG repeats; however, instability in the normal range has also been documented (Nolin et al., 2013). The full mutation occurs only when it is transmitted from the mother; and it is rare for a contraction to occur when the unstable expanded allele is passed on by the mother (Lozano et al., 2016). Here, we described a rare case of prenatal diagnosis of male fetus that inherited an allele that contracted to a normal repeat size from the mother with the full mutation.

## Materials and Methods

Subjects: Written informed consent was obtained from the studied subjects for publication of this manuscript.

Allele categories were as described in Maddalena et al. (2001) (normal 5–54 CGG, premutation alleles 55–200 CGG repeats, full mutation alleles >200 CGG repeats).

A 40-year-old full mutation woman known to carry size mosaicism for the presence of a premutation and a full mutation alleles (29, 160, >200 CGG repeats) in the *FMR1* gene was referred to our laboratory for prenatal diagnosis of her 24-week fetus. It was a spontaneous pregnancy after a long history of failure in preimplantation genetic diagnosis (PGD) pregnancies. The laboratory received 30 ml of amniotic fluid, which were divided into two different syringes, and 5 ml was used for the direct DNA extraction while the remaining amount was cultured for karyotyping and DNA extraction.

### Amniocytes Culture

The amniocytes were centrifuged and half of the pellet suspended in CHANG medium (Irvine Scientific, Santa Ana, CA, United States) while the other half was suspended in BioAMF-2 (Biological Industries, Beit-HaEmek, Israel) and cultured in 25 cm flasks for 10–14 days. When cells reached confluence, they were either harvested for DNA extraction or trypsinized for the next passage. Cells harvested at the fifth and at seventh; by the eighth passage, the culture failed.

### Genomic DNA Extraction

Genomic DNA (gDNA) was extracted from peripheral blood samples collected from the mother, the grandmother, and from the father using QIAsymphony DNA Midi Kit (96)-931255 and the QIAsymphony machine according to the manufacturer’s instructions. Isolation of gDNA from cultures cells was carried out using the QIAamp DNA blood Mini Kit-51104 (Qiagen, Hilden, Germany) and the QIAsymphony machine according to the manufacturer’s instructions. DNA was extracted from amniotic fluid using the QIAamp DNA blood Micro Kit-56304 (Qiagen, Hilden, Germany), manually or using the QIAcube machine according to the manufacturer’s instructions.

### Triple-Primed FXS PCR

Genomic DNA (gDNA) (40–60 ng) was amplified with the Amplidex *FMR1* PCR assay (Asuragen, Austin, TX, United States) as previously described (Nahhas et al., 2012) and accordingly to the manufacturer’s instructions. Samples were analyzed by the 3130xl Genetic Analyzer (Applied Biosystems Inc., ABI, Foster City, CA, United States) and electropherograms were analyzed using GeneMapper 4.0 (4.1 for 3500xL data) (Filipovic-Sadic et al., 2010).

### *FMR1* 5′ UTR Sequencing

Genomic DNA (gDNA) was amplified in 20 μl reaction using forward primer FMR-C: 5′-GCTCAGCTCCGTTTCGGTTTCACTTCCGGT-3′ and reverse primer FMR-F: 5′-AGCCCCGCACTTCCACCACCAGCTCCTCCA-3′ (SIGMA). PCR products were sequenced with BigDye Terminator v1.1 Cycle Sequence Kit – Applied Biosystems – chemistry on a 3130xl Genetic Analyzer (Life Technologies). Electropherograms were analyzed using GeneMapper 4.0 (4.1 for 3500xL data).

### Haplotype Analysis

Maternity studies were performed using Elucigene QSTR plus V2 kit. A segregation study was performed on the grandmother, the mother, and the fetus using the following polymorphic makers: DXS106, DXS8028, DXS998, *FMR1:* 146993469–147032647, DXS106, DXS8069, DXS8061, DXS1073: and florescent primers (Richards et al., 1991; Verkerk et al., 1993). Alleles were resolved on capillary gel electrophoresis using ABI 3130xl apparatus. Electropherograms were analyzed using GeneMapper 4.0 (4.1 for 3500xL data).

### Methylation Analysis

Methylation analysis was carried out using the Amplidex *FMR1* mPCR *FMR1* kit (Asuragen, Austin, TX, United States) as previously described by Chen et al. (2014). Briefly, two aliquots of 40 ng gDNA isolated from the fetus, from a methylated male control and from a normal male were prepared for methylation assessment. One aliquot was mixed with 4 μL Digestion Enzyme Mix (containing HhaI and AvaI), and the other with 4 μL Digestion Control Mix (containing digestion buffer). Aliquoted samples were incubated at 37°C overnight. Following digestion, aliquots from each sample analyzed according to Asuragen’s instruction. The results were analyzed using the 3130xl Genetic Analyzer (Applied Biosystems Inc., ABI, Foster City, CA, United States). Electropherograms were analyzed using GeneMapper 4.0 (4.1 for 3500xL data) as described by Filipovic-Sadic et al. (2010).

## Results

**FIGURE 1:**
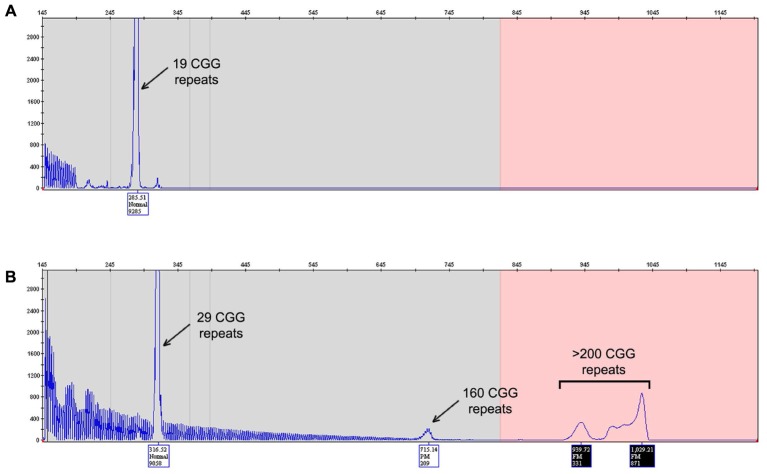
*FMR1* mutation – length test. Electropherogram of the Asuragen test results showing the fetus **(A)** and the mother **(B)** CGG repeats length results. The fetus results show presence of a pronounced peak at 19 CGG repeats **(A)** and the mother results **(B)** it shows normal allele with 29 CGG repeats and the unstable allele with mosaicism, a premutation allele of 160 CGG repeats and full mutation greater than 200 CGG repeats.

Capillary electrophoresis analysis of two different direct amniocentesis samples of DNA revealed the presence of two peaks: one less abundant [3000 relative fluorescent units (RFU)] corresponding to 29 CGG repeat allele and the other much more abundant peak (9000 RFU) corresponding to 19 CGG repeat allele (**Figure 1**). Karyotype analysis confirmed a male fetus.

DNA from eight amniocyte cultures was also tested: two directly from cultured amniocytes, two from the first passage, two from the fifth passage, and two from the seventh passage. All samples showed presence of only the 19 CGG repeat allele as observed in DNA isolated from the amniotic fluid but without the less abundant peak corresponding to the 29 CGG repeat allele. The less abundant peak observed from DNA isolated from amniotic fluid points to low level of mother contamination, which can been detected by the Amplidex PCR (Asuragen) as it has been proven to be much more sensitive than other techniques (Filipovic-Sadic et al., 2010; Tassone, 2015). None of the samples tested showed evidence of an expanded allele excluding the possibility of mosaicism.

Sequence analysis confirmed the presence of a 19 CGG repeat allele in both DNA isolated from amniotic fluid and DNA isolated from cultured amniocytes after seven passages (**Figure 1**). No AGG interruptions were observed within the allele.

**FIGURE 2:**
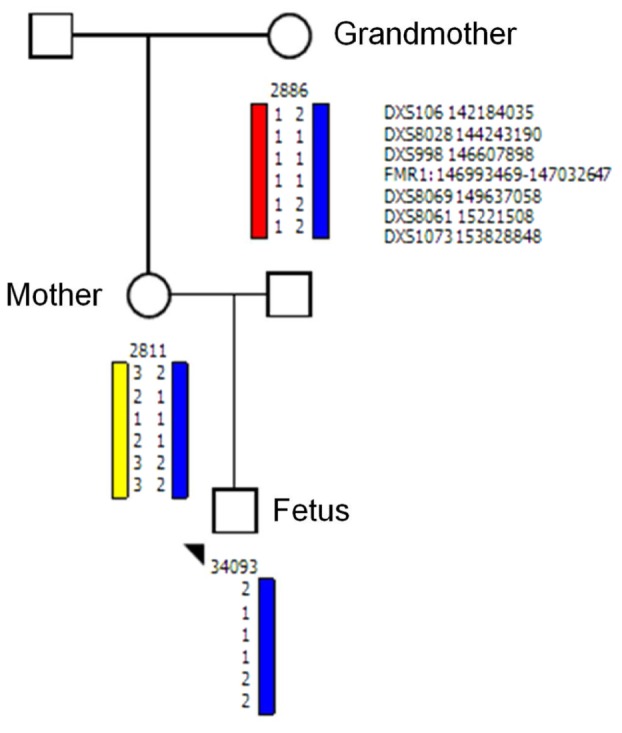
Haplotype analysis for allele discrimination and tracing the origin of *FMR1* fetal allele. Haplotype analysis using the polymorphic makers: DXS106, DXS8028, DXS998, *FMR1*: 146993469–147032647, DXS106, DXS8069, DXS8061, DXS1073 shows that the unstable allele transmitted from the grandmother to the mother and to her fetus.

Testing the origin of the *FMR1* allele by haplotype analysis using the DNA derived from the mother, the fetus, and from the maternal grandmother showed that the unstable allele was transmitted from the grandmother to her daughter who transmitted it to her male fetus; thus confirmed maternity (**Figure 2**).

Methylation assay of the *FMR1* gene using AvaI and HhaI showed no methylation in the fetus cells while it showed a normal methylation in both *FMR1* alleles of the mother suggesting that methylation assay is not reliable for fetal cells.

## Discussion

The case described here represents a rare case of transmission from a full mutation mother to her offspring with an allele pattern different from what we would have expected in such cases. It is a prenatal diagnosis case of a male fetus that inherited an allele with a CGG repeats number in the normal size range (19 CGG repeats) derived from the unstable expanded maternal allele (160, >200 CGG repeats). It is well known that the majority (near to 100%) of the male fetuses that inherited unstable *FMR1* allele from mosaic mothers are expected to inherit a full mutation allele. Contraction of a maternal high premutation (>100 CGG repeats)/full mutation *FMR1* allele during transmission has been rarely described (Yrigollen et al., 2014; Miranda et al., 2015; Tabolacci et al., 2016; Maia et al., 2017).

To our knowledge this is the first report of a prenatal diagnosis case showing a pattern of contraction of an expanded *FMR1* unstable allele to a normal size allele. Tabolacci et al. (2016) previously described a case of a healthy 10-year-old boy who inherited a contracted unstable *FMR1* allele from his mother. The authors described a family with a carrier mother (190 CGG repeats) and her three children. Two of them presented with FXS and had expanded alleles in the full mutation range (265–830 and 430–790 CGG repeats, respectively). In contrast, the healthy boy inherited a contracted allele of 43 CGG repeats. Another study (Maia et al., 2017) described four FXS boys carrying mosaic alleles ranging from the normal (35, 26, 39, and 18 CGG repeats) to the full mutation range (>200 CGG repeats). These studies sharpen the obscure mechanism of expansion of the trinucleotide repeat in the *FMR1* gene, indicating that the same allele during the generation might expand or contract and possibly undergo methylation.

Since this is a prenatal diagnosis case, the questions are as follows: Is the unstable allele contracted to the normal range able to become a stable normal allele with no phenotypic consequences? Is it a normal unstable allele that may lead to some clinical involvement? Will it expand during transmission in the following generations? Is it a case of mosaicism through an undetected expanded allele of the *FMR1* gene, which may cause features of FXS?

It is of importance to understand the consequence of contraction of high premutation/full mutation *FMR1* allele as understanding the mechanism of *FMR1* instability can improve genetic counseling of the family.

Although CGG instability within the *FMR1* gene occurs mainly from the premutation to the full mutation range, instability on transmission with CGG repeat length, defined as any measurable repeat change from the parent to the offspring, has also been described in allele within the normal range (Nolin et al., 2013). The magnitude of instability correlates to the presence of AGG interruptions (Nolin et al., 2013; Yrigollen et al., 2014). Normal alleles have an AGG interruption that usually occurs after 9 or 10 CGG triplets (Yrigollen et al., 2014). However, it has been found that increased instability seems to be correlated with the presence of the first interruption at the 10th triplet (Gunter et al., 1998) and to the total number of AGGs. The loss of AGGs occurs in a polarized way at the 3′-end, creating a long pure (CGG)n with higher mutability (Kunst and Warren, 1994; Limprasert et al., 2016). In the presented case, the allele was a pure stretch of CGG without any AGG interruption. The AGG interspersions in the CGG repeat region are well known as an important element for stability of the repeats (Ennis et al., 2007; Fernandez-Carvajal et al., 2009; Carolyn et al., 2012; Nolin et al., 2013, 2015; Yrigollen et al., 2014).

Sharony et al. (2012), Wakeling et al. (2014), and also our laboratory (data not shown) experienced instability/mosaicism in the normal–intermediate range (less than 55 CGG repeats). Wakeling et al. (2014) reported that about 0.4% of the normal population tested showed an extra allele. Our experience from the routine *FMR1* screening test of normal population as well as the Sharony et al. (2012) study showed a lower frequency of cases with an extra allele (12 out of 13,000 cases; data not shown) in the normal range size. The presence of an extra allele could be attributed to the presence of a cryptic duplication event of the *FMR1* locus (Mononen et al., 2007; Vengoechea et al., 2012) of an early postzygotic expansion or contraction of the *FMR1* repeat tract or to a somatic instability of the CGG tract. While somatic instability of premutation and full mutation alleles is well documented (Cambouris et al., 1996; Dobkin et al., 1996; Mingroni-Netto et al., 1996), information regarding unstable normal alleles (Sullivan et al., 2005; Nolin et al., 2011) is limited. In contrast to the report of Wakeling et al. (2014), Maia et al. (2017) reported on four FXS mosaic males carrying normal, premutated and full mutation *FMR1* alleles. The authors suggested that the normal allele of these patients resulted from postzygotic contraction of the full expansion. The authors study suggested the existence of a predisposing haplotype in the population, which they named “risk lineage-specific haplotype,” more prone to large contraction.

Although the mechanism(s) of the *FMR1* instability are still unknown, a mice model including the interplay among components of the base excision repair, mismatch repair, and transcription coupled repair pathways has been proposed (Zhao et al., 2016). The authors indicated that expanded alleles are unstable and can undergo to an expansion or contraction event through different mechanisms. Contraction events have been observed on both paternal and maternal transmission although more frequently on paternal transmission (Nolin et al., 2015). Although *FMR1* expansion in human occurred almost exclusively through maternal inheritance, there is no reference to any sex factor. Contractions may derive from simple strand slippage during replication or repair; however, to date, which factors are involved in the processes of repeat instability at the *FMR1* locus remain an unanswered question.

Although PCR-based approach and the sequencing tests used in the present study have shown to be very reliable, we cannot categorically exclude the presence of an expanded allele. However, the results demonstrate the presence of a normal size allele with no mosaicism, most likely leading to a normal phenotype as the case described by Tabolacci et al. (2016).

Our hypothesis is that the maternal premutation/full mutation allele contracted pre-zygotically either in the mitotic or in the meiotic stage resulting in a normal size allele in the fertilized egg with no syndromic consequence.

## Ethics Statement

All subjects gave written informed consent in accordance with the Declaration of Helsinki.

## Author Contributions

EM: Performed design for this study, management, and wrote the manuscript. AJ: Performed Fragile tests and Fragile methylation. NM: Performed Haplotype testing and confirmed the results. AK: Performed medical management and clinical evaluation of the case and the family. RH: Worked as a part of Genetic advisory and edited the article. FT: Performed the evaluation of the results and edited the article.

## Conflict of Interest Statement

The authors declare that the research was conducted in the absence of any commercial or financial relationships that could be construed as a potential conflict of interest.
